# Associations between breastfeeding and cognitive function in children from early childhood to school age: a prospective birth cohort study

**DOI:** 10.1186/s13006-020-00326-4

**Published:** 2020-09-29

**Authors:** Kyoung Min Kim, Jae-Won Choi

**Affiliations:** 1 Department of Psychiatry, Dankook University Hospital, Cheonan, Republic of Korea; 2 Department of Psychiatry, Dankook University College of Medicine, 119 Dandae-ro, Dongnam-gu, Cheonan, Chungnam-do 31116 Republic of Korea; 3 Department of Psychiatry, Gyeongsang National University Hospital, 79, Gangnam-ro, Jinju-si, Gyeongsangnam-do 52727 Republic of Korea

**Keywords:** Breastfeeding, Cognitive development, Childhood, School-age children

## Abstract

**Background:**

Despite evidences of breastfeeding for preventing acute physical illnesses in infants, the evidence for the association between breastfeeding and long-term cognitive development is not yet convincing.

**Methods:**

The data of nationwide representative sample of 1752 children born between 2008 and 2009 in Korea were prospectively assessed from the fetal period to examine the benefits of breastfeeding and cognitive development. Breastfeeding duration was prospectively assessed by parents. The Korean Ages and Stages Questionnaire and the Korean version of Denver II were used to assess early development annually from 5.5 to 26.2 months of age. Language development at 3 years of age was assessed with Receptive and Expressive Vocabulary Tests. Cognitive function at 8 years of age was assessed using multifactorial intelligence test.

**Results:**

In the analysis of categorical variables, children who were breastfed for > 1 and ≤ 3 months displayed significantly higher odds ratios for delayed development assessed with Korean Ages and Stages Questionnaire at 14.1 months than those breastfed for > 3 and ≤ 6 months (OR = 2.21; 95% CI: 1.08, 4.50), but no significant differences in other rounds of assessments. In the analysis with continuous variables, there were significant differences among six groups of breastfeeding duration in communication (F = 3.72; *p* < 0.002) and problem solving (F = 3.09; *p* < 0.009) at 14.1 months, expressive language (F = 3.74; *p* = 0.002) at 3 years, and calculation (F = 2.43; *p* < 0.033) at 8 years. When analyzed by two groups, children breastfed for > 3 months scored significantly higher on the communication (F = 17.71; *p* < 0.001) and problem-solving (F = 11.26; *p* < 0.001) subscales at 14.1 months, and expressive language (F = 12.85; *p* < 0.001) at 3 years, and vocabulary (F = 6.78; *p* = 0.009) and language inference (F = 5.62; *p* = 0.018) at 8 years, compared to children breastfed for 3 months or less.

**Conclusion:**

We found that cognitive development was improved in children that were breastfed for > 3 months. Although these results are supported by previous studies, it is important to note that other factors were reported as larger determinants of cognitive development than breastfeeding. Future studies that examine the underlying mechanism for the association between breastfeeding and cognitive development are warranted.

## Background

Breastfeeding is an important component of nutrition for infants and it provides various health benefits to the child and mother [1]. Breastfeeding has clear short-term benefits for reducing morbidity and mortality from infectious disease in infants [2]. Breastfeeding provides health benefits and prevention of acute physical illnesses including gastrointestinal illnesses, otitis media, respiratory tract infections, and neonatal necrotizing enterocolitis to infants [1, 3]. Breastfeeding may also prevent infants from developing chronic diseases such as asthma, allergies, and obesity [1].

Cognitive development in children has been another effect examined in breastfeeding research. The topic was first studied by Hoefer and Hardy in 1929 and multiple other studies have since examined the associations between breastfeeding and cognitive function of children with consistently reported positive associations [4]. A meta-analysis of 11 studies reported that infants who were breastfed had higher intelligence quotients (IQ) by 5.32 points (unadjusted) and 3.16 points when adjusted for covariates [5]. In addition, the higher levels of cognitive function observed in breastfed infants were stable across successive ages. A more recent meta-analysis of 17 studies on the relationship between breastfeeding and intelligence reported that breastfed subjects presented a higher IQ by 3.44 points or by 2.62 points when controlled for maternal IQ [6].

A randomized experiment performed with consideration of the concerns raised regarding previous observational studies also reported significantly higher verbal IQ, performance IQ, and full-scale IQ in the breastfed group by 7.5, 2.9 and 5.9 points, respectively [7]. The breastfed group also scored higher in teacher ratings of both reading and writing. Likewise, cross-population studies of British and Brazilian cohorts reported that longer breastfeeding duration was related to higher IQ scores by 3–6 points [8]. The cognitive benefits of breastfeeding were reported to persist into adulthood. IQ scores at the age of 30 were 3.76 points higher in participants who were breastfed for ≥12 months compared to those who were breastfed for < 1 month [9].

Despite the evidence for the positive associations between breastfeeding and cognitive development by multiple studies within various populations, there are few studies that have employed multiple assessment tools and repetitive assessment of cognitive development at multiple points. In addition, previous studies in the Korean population, especially using prospective methods, are sparse. A study of the Korean population reported significantly higher IQ by 4.07 points in breastfed children compared to non-breastfed children assessed at age of 9 years [10]. Kim et al. reported that breastfed Korean children had significantly higher learning quotient scores in speaking, reading, writing, spelling, and mathematical calculation than children who were never-breastfed [11]. However, these studies were based on a cross-sectional sample and retrospective information on breastfeeding; therefore, the causal relationship between breastfeeding and learning skills cannot be drawn and recall bias is possible in these studies. The present study aims to examine the associations between breastfeeding and cognitive development in Korean children from ages of 1–8 years using multimodal and multi-informant assessment and a prospective study design.

## Methods

### Participants

The present study utilized data collected from the Panel Study on Korean Children (PSKC). The PSKC is an ongoing longitudinal panel study conducted by the Korea Institute of Child Care and Education since 2008. The participants in PSKC were invited by stratified multistage sampling using resident registration data to represent all nationwide household populations. A total of 2150 children born between 2008 and 2009 in Korea were enrolled in the study from the fetal period and evaluated prospectively for breastfeeding and cognitive development at 5.5 (T1), 14.1 (T2), 26.2 (T3), 38.7 (T4), and 99.2 (T9) months of age. Because of the challenges of longitudinal cohort studies, there was some missing data for the follow-up assessments. In our study, we analyzed data collected from 1752 children whose assessments of breastfeeding and K-ASQ at T3 (26.2 months) were present.

### Measurements

Demographic variables including the child’s sex, age, gestational period, birth weight, parents’ education level, and household income were assessed by paper and pencil interviews and computer-assisted personal interviews. Breastfeeding data from T1 (5.5 months) to T4 (38.7 months) was prospectively collected by computer-assisted personal interviews.

#### Early development

To assess early cognitive development at T1 (5.5 months), T2 (14.1 months), and T3 (26.2 months), the participants were assessed using the Korean Ages and Stages Questionnaires (K-ASQ) and the Korean version of Denver II, which are widely used screening tools for early development. K-ASQ is a screening tool for the developmental progress of infants and toddlers as rated by parents [12]. The K-ASQ comprised 30 items rated on three-point Likert scales under the following five subdomains: communication, gross motor, fine motor, problem-solving, and personal–social [13]. Scores that were two standard deviations below the average in each subdomain were coded as atypical. Denver II is another screening tool for early development with a validated Korean version [14, 15]. Denver II codes the development of children to the dichotomous outcomes of “normal” or “suspicious” based on the assessment scores. Denver II comprised four subdomains: personal–social, fine motor/adaptive, language, and gross motor. K-ASQ and Denver II could not be included because of copyright; however, additional information regarding both tools can be obtained from the Panel Study on Korean Children website [16].

#### Cognitive function in middle childhood and school age

Language abilities at T4 (38.7 months) were assessed by the Receptive and Expressive Vocabulary Test (REVT), which is comprised of 185 Korean vocabulary items and two subscales of receptive and expressive language tests [17]. The REVT results were coded to the percentile scores of 1 (< 10%) to 11 (100%) as a continuous variable and “Normal or mildly delayed” and “Markedly delayed” as binary categorical variables. The cognitive function of school-age children at T9 (99.2 months) was assessed in terms of intelligence and academic performance. The intelligence of children was assessed using the multifactorial intelligence test (M-FIT). The M-FIT is comprised of six subdomains (vocabulary, language inference, schematization, calculation, spatial perception, and reasoning), each with 20 item tests. The scores of M-FIT are presented with the T-score and percentile score (0–100) based on normative data. Our analysis used the T-score, which is a norm-referenced standardized score with a mean of 50 points and standard deviation of 10 points. The T-score of ≤37 on each subscale was labeled as “delayed,” and the participants with the “delayed” scores on at least one subscale were categorized as “delayed” for the categorical analysis.

### Statistical analysis

Although the criterion for breastfeeding duration to group participants vary widely in previous studies, many studies included “never,” “1 month,” “3 months,” “6 months,” and “12 months” as the duration criteria [6]. In our study, participants were grouped by the following breastfeeding durations according to previous studies [9, 18]: “never,” “up to 1 month,” “1–3 months,” “3–6 months,” “6–12 months,” “12–18 months” and “over 18 months.” We used logistic regression to investigate the odds ratio for delayed development of the early period (T1, 5.5 months to T3, 26.2 months) assessed by Denver II and K-ASQ. To compare the outcomes of K-ASQ as continuous variables, language ability at T4 (38.7 months), and intelligence and academic function at T9 (99.2 months) among the groups of breastfeeding duration, analysis of variance (ANOVA) and analysis of covariance (ANCOVA) were utilized. In all analyses, the adjusted model included the children’s sex, age, gestational age, birth weight, parents’ education level, and household income as covariates. To adjust for multiple comparisons included in our analysis, we performed the Benjamini–Hochberg test with a false discovery rate threshold of 0.05 for the crude and adjusted models, respectively [19]. Statistical analyses were conducted using the software package SPSS 25.0 for Windows (IBM Co., Armonk, NY, USA).

### Ethics

We provided parents with information on the purpose and procedure of the study and written informed consent was obtained from parents before enrollment. This study was performed in accordance with the ethical standards laid down in the 1964 Declaration of Helsinki and its later amendments. The study protocol was approved by the Institutional Review Board of the Korean Institute of Child Care of Education (KICCEIRB-2016-07).

## Results

### Demographic characteristics and prevalence of breastfeeding

Among the 2150 children enrolled in the study, 1752 children, whose assessment findings for breastfeeding and K-ASQ at the age of 14 months were available, were included in the final analysis. Among them, 1632, 1704, 1752, 1630, and 1398 children were included in the analysis of each assessment wave at 5.5 (T1), 14.1 (T2), 26,2 (T3), 38.7 (T4), and 99.2 (T9) months of age, respectively, because of some missing assessments and dropouts at follow-up.

The demographic characteristics of participants are presented in Table 1 and the geographical distribution of participants is presented in Table S1. Of the total participants, 893 (51%) children were male, 52 (3.0%) children were born preterm and 49 (2.8%) children were born with low birth weights. The mean T1 household income of participants included in the analysis was 3193 (SD = 1462) thousand KRW. It is similar to the Korean national household income level (mean = 3390 thousand KRW) in 2008 [20] and is not significantly different from the income of the excluded participants (mean = 3299; SD = 1653 thousand KRW; F = 1.35; *p* = 0.245). Moreover, the regional distribution of included participants was not significantly different from the excluded participants (Table S1). These findings indicate that our sample was not susceptible to the selection and attrition bias.

**Table 1:** Demographic characteristics of the participants

Variables	n (%)
Sex	
Male	893 (51.0)
Female	859 (49.0)
Gestational age (days)	
< 259 (preterm)	52 (3.0)
≥ 259 (term)	1638 (93.5)
Unknown	62 (3.5)
Birth weight (g)	
< 2500	49 (2.8)
≥ 2500	1647 (94.0)
Unknown	56 (3.2)
Household income	
≤ 2000	432 (24.7)
> 2000 and ≤ 3000	532 (30.4)
> 3000 and ≤ 4000	315 (18.0)
> 4000 and ≤ 5000	193 (11.0)
> 5000	111 (6.3)
Unknown	169 (9.6)
Paternal educational level (years)	
≤ 12	520 (29.7)
> 12 and < 16	486 (27.7)
≥ 16	680 (38.8)
Unknown	66 (3.8)
Maternal educational level (years)	
≤ 12	454 (25.9)
> 12 and < 16	362 (20.7)
≥ 16	809 (46.2)
Unknown	127 (7.2)
Age at each assessment (months; mean; SD)	
T1	5.5 (1.2)
T2	14.1 (1.0)
T3	26.2 (1.3)
T4	38.7 (1.4)
T9	99.2 (1.4)

The prevalence and duration of breastfeeding are presented in Table 2. The proportion of children who were ever-breastfed in our study was 97.4%. The proportion of never-breastfed children was 2.6% and children who were breastfed for ≤1 month was 15.8%. The proportion of children who continued breastfeeding after 6 months was 61.8%.

**Table 2:** Proportion of the participants in each breastfeeding duration category

	n	%
Never breastfed	45	2.6
≤1 month	231	13.2
> 1 month to ≤3 month	281	16.0
> 3 months to ≤6 months	205	11.7
> 6 months to ≤12 months	445	25.4
> 12 months or ≤ 18 months	381	21.7
> 18 months	164	9.4
Total	1752	100.0

### Odds ratios for delays in development based on the duration of breastfeeding

The odds ratio for delayed development at T1 (5.5 months) to T3 (26.2 months) are presented in Table 3. In the six group comparison, odds ratios for delayed development assessed with K-ASQ at T2 (14.1 months) were significantly higher in children breastfed for 1–3 months by 2.21 (95% CI 1.08, 4.50; crude) or 2.63 (95% CI 1.20, 5.77; adjusted) folds, compared to the reference group (children breastfed for 3–6 months). The comparison of two groups at T3 (26.2 months) presented significantly higher odds ratios for delayed development by 1.45-fold (95% CI 1.02, 2.07; crude) in children breastfed for ≤3 months than those breastfed for > 3 months. In some development assessments (i.e., Denver II at T1 and T3), children breastfed for ≤1 month presented lower odds ratios for development delay. However, these odds ratios did not reach significance. The odds ratio for language development delay at T4 showed no significant differences among the six groups. Although children breastfed for ≤3 months showed significantly higher odds ratio for delay in expressive language by 1.64 folds (95% CI 1.17, 2.30; crude), its significance was lost after adjustment. In the performance of intelligence at T9, children breastfed for > 18 months revealed a higher odds ratio (2.54; 95% CI 1.09, 5.90; crude model) for delayed development compared with children breastfed for 3–6 months.

**Table 3:** Odds ratio for delayed development at T1, T2, and T3 based on the duration of breastfeeding

Breastfeeding duration (months)	0 to ≤1	> 1 and ≤ 3	> 3 and ≤ 6	> 6 and ≤ 12	> 12 and ≤ 18	> 18	Two groups^a^
Crude (n)							
Denver II at T1 (*n* = 1429)	0.67 (0.32, 1.44)	1.07 (0.53, 2.15)	1.00	1.16 (0.62, 2.19)	0.89 (0.46, 1.73)	0.88 (0.39, 1.99)	0.86 (0.57, 1.30)
Denver II at T2 (*n* = 1698)	0.85 (0.55, 1.30)	1.04 (0.68, 1.58)	1.00	0.88 (0.60, 1.30)	0.98 (0.66, 1.46)	0.91 (0.56, 1.48)	1.00 (0.79, 1.27)
Denver II at T3 (*n* = 1731)	0.71 (0.39, 1.30)	0.98 (0.56, 1.72)	1.00	0.84 (0.49, 1.42)	0.76 (0.44, 1.32)	1.23 (0.66, 2.26)	0.95 (0.68, 1.32)
K-ASQ at T1 (*n* = 1632)	1.01 (0.42, 2.45)	1.41 (0.62, 3.24)	1.00	0.88 (0.38, 2.01)	1.28 (0.57, 2.85)	1.14 (0.43, 3.04)	1.14 (0.72, 1.81)
K-ASQ at T2 (*n* = 1704)	2.02 (0.98, 4.15)	**2.21 (1.08**, 4.50)*	1.00	1.54 (0.77, 3.09)	1.94 (0.97, 3.87)	2.12 (0.97, 4.64)	1.28 (0.92, 1.79)
K-ASQ at T3 (*n* = 1752)	1.73 (0.88, 3.43)	1.51 (0.75, 3.01)	1.00	1.26 (0.65, 2.44)	0.99 (0.49, 1.99)	1.17 (0.52, 2.63)	**1.45 (1.02**, 2.07)*
REVT-E at T4 (*n* = 1630)	1.62 (0.88, 2.99)	1.27 (0.67, 2.38)	1.00	0.95 (0.52, 1.73)	0.91 (0.49, 1.71)	0.48 (0.19, 1.19)	**1.64 (1.17**, 2.30)*
REVT-R at T4 (*n* = 1625)	1.29 (0.53, 3.13)	1.67 (0.71, 3.92)	1.00	1.43 (0.63, 3.22)	1.25 (0.54, 2.91)	0.91 (0.31, 2.69)	1.21 (0.77, 1.88)
M-FIT at T9 (n = 1752)	1.05 (0.59, 1.88)	0.85 (0.47, 1.54)	1.00	0.69 (0.39, 1.21)	0.87 (0.50, 1.52)	**1.94 (1.07**, 3.50)*	0.99 (0.71, 1.38)
Adjusted (n)							
Denver II at T1 (*n* = 1264)	0.58 (0.26, 1.30)	0.82 (0.39, 1.76)	1.00	1.10 (0.56, 2.15)	0.75 (0.37, 1.53)	0.66 (0.26, 1.65)	0.77 (0.49, 1.22)
Denver II at T2 (*n* = 1455)	1.07 (0.66, 1.73)	1.24 (0.77, 1.98)	1.00	1.10 (0.71, 1.71)	1.31 (0.84, 2.05)	1.08 (0.63, 1.86)	1.01 (0.77, 1.31)
Denver II at T3 (*n* = 1472)	0.54 (0.27, 1.09)	0.96 (0.52, 1.79)	1.00	0.86 (0.48, 1.54)	0.87 (0.48, 1.57)	1.27 (0.65, 2.49)	0.79 (0.54, 1.16)
K-ASQ at T1 (*n* = 1436)	1.40 (0.46, 4.21)	2.09 (0.73, 6.00)	1.00	1.38 (0.48, 3.97)	1.97 (0.70, 5.53)	1.63 (0.48, 5.54)	1.14 (0.67, 1.93)
K-ASQ at T2 (*n* = 1461)	1.95 (0.86, 4.42)	**2.63 (1.20**, 5.77)*	1.00	1.63 (0.75, 3.53)	2.03 (0.94, 4.38)	**2.54 (1.09**, 5.90)*	1.30 (0.90, 1.88)
K-ASQ at T3 (*n* = 1491)	1.64 (0.76, 3.52)	1.61 (0.75, 3.45)	1.00	1.40 (0.67, 2.93)	1.20 (0.56, 2.56)	1.10 (0.44, 2.77)	1.33 (0.89, 1.99)
REVT-E at T4 (*n* = 1397)	1.37 (0.70, 2.69)	0.98 (0.49, 1.98)	1.00	0.96 (0.50, 1.84)	0.93 (0.47, 1.82)	0.44 (0.17, 1.19)	1.33 (0.91, 1.93)
REVT-R at T4 (*n* = 1394)	1.83 (0.63, 5.31)	2.43 (0.87, 6.79)	1.00	2.03 (0.74, 5.53)	1.91 (0.68, 5.33)	1.21 (0.34, 4.33)	1.26 (0.78, 2.02)
M-FIT at T9 (n = 1491)	0.85 (0.45, 1.61)	0.78 (0.40, 1.50)	1.00	0.68 (0.37, 1.27)	0.81 (0.43, 1.50)	**1.95 (1.02**, 3.73)*	0.87 (0.60, 1.27)

^a^Odds ratio for development delay in children breastfed for ≤3 months compared with children breastfed for more than 3 months

Adjusted model included children’s sex, age, gestational age, birth weight, parental educational level, and household income level as covariates

REVT-E: receptive and expressive vocabulary test (expressive); REVT-R; receptive and expressive vocabulary test (receptive);

M-FIT: multifactorial intelligence test; * *p* < 0.05

### Comparison of cognitive function scores based on the duration of breastfeeding

The comparison of scores for each cognitive function test as a continuous variable are presented in Table 4 and Table S2. The subscales of communication (*F* = 3.72; *p* = 0.002; only in crude model) and problem-solving (*F* = 3.09; *p* = 0.009) at T2 (14.1 months) differed significantly among the six groups of breastfeeding duration, with a higher performance in children breastfed > 3 months than children breastfed for ≤3 months. The scores on K-ASQ at T1 and T3 among the six groups did not differ significantly.

**Table 4:** Comparison of scores on intellectual function tests among the six groups of breastfeeding duration

Breastfeeding duration(months)	0 to ≤1	> 1 and ≤3	> 3 and ≤6	> 6 and ≤ 12	> 12 and ≤ 18	> 18	Crude			Adjusted		
							F	*df*	p	F	*df*	p
K-ASQ at T1												
Communication	54.0 (8.5)	53.4 (9.2)	53.1 (9.4)	53.2 (9.1)	54.7 (8.0)	54.4 (8.8)	1.65	5, 1626	0.144	0.77	5, 1414	0.574
Fine motor	54.1 (9.1)	54.4 (8.9)	55.2 (8.6)	55.0 (8.5)	55.0 (8.2)	54.3 (10.0)	0.67	5, 1626	0.644	0.47	5, 1414	0.799
Gross motor	57.5 (6.3)	57.1 (6.9)	56.6 (7.5)	57.6 (5.7)	57.9 (5.4)	57.3 (7.2)	1.34	5, 1626	0.243	0.97	5, 1414	0.436
Personal, social	54.6 (7.7)	53.9 (8.6)	53.4 (9.9)	55.2 (7.4)	55.1 (7.6)	55.3 (8.4)	2.20	5, 1626	0.052	0.90	5, 1414	0.477
Problem solving	56.4 (6.5)	55.5 (8.0)	55.9 (7.9)	55.9 (7.3)	56.4 (7.0)	56.3 (7.7)	0.65	5, 1626	0.662	0.77	5, 1414	0.571
K-ASQ at T2												
Communication	47.9 (12.1)	47.6 (11.8)	49.6 (10.9)	50.2 (10.5)	50.5 (10.8)	50.0 (11.1)	3.72	5, 1698	0.002**^, a^	1.84	5, 1439	0.102
Fine motor	47.7 (11.8)	46.4 (12.4)	48.6 (10.9)	47.7 (12.0)	48.7 (11.7)	47.5 (12.1)	1.38	5, 1698	0.228	1.67	5, 1439	0.140
Gross motor	56.4 (8.8)	56.3 (9.5)	57.2 (7.0)	57.2 (7.7)	56.0 (9.6)	55.2 (10.6)	1.64	5, 1698	0.146	2.05	5, 1439	0.069
Personal, social	51.4 (11.9)	52.7 (10.2)	52.1 (10.9)	52.5 (10.9)	53.1 (10.8)	52.3 (11.0)	0.84	5, 1698	0.518	0.84	5, 1439	0.518
Problem solving	48.6 (13.4)	47.5 (13.1)	49.5 (11.9)	50.6 (10.7)	50.5 (11.1)	49.0 (12.2)	3.09	5, 1698	0.009**	3.83	5, 1439	0.002**^, a^
K-ASQ at T3												
Communication	52.0 (11.1)	51.8 (11.6)	52.9 (11.1)	53.5 (10.8)	53.7 (10.5)	52.4 (11.1)	1.63	5, 1746	0.148	1.48	5, 1466	0.192
Fine motor	52.7 (9.4)	52.6 (10.2)	53.2 (8.6)	53.4 (8.2)	53.7 (8.3)	54.1 (9.1)	1.02	5, 1746	0.401	0.54	5, 1466	0.742
Gross motor	57.6 (6.3)	56.8 (7.1)	57.5 (5.4)	57.4 (5.5)	57.1 (5.9)	57.3 (5.5)	0.62	5, 1746	0.685	1.04	5, 1466	0.390
Personal, social	54.7 (9.3)	54.0 (9.7)	54.7 (8.4)	55.1 (8.4)	55.1 (8.4)	55.4 (9.1)	0.79	5, 1746	0.559	0.29	5, 1466	0.921
Problem solving	53.5 (8.0)	53.2 (8.4)	55.0 (7.1)	54.1 (7.9)	54.7 (7.2)	54.1 (8.4)	1.89	5, 1746	0.093	1.07	5, 1466	0.375
REVT at T4												
Expressive	4.2 (3.1)	4.5 (3.1)	4.6 (3.1)	5.2 (3.3)	5.0 (3.3)	4.9 (3.2)	3.74	5, 1624	0.002**^, a^	2.44	5, 1373	0.033*
Receptive	6.4 (3.6)	6.5 (3.6)	6.3 (3.5)	6.5 (3.6)	6.6 (3.6)	6.3 (3.5)	0.21	5, 1619	0.958	0.40	5, 1370	0.846
M-FIT at T9												
Vocabulary	55.4 (10.7)	55.4 (10.3)	57.6 (10.4)	56.8 (10.3)	57.3 (10.5)	56.1 (11.6)	1.75	51,392	0.120	0.63	5, 1176	0.678
Language inference	56.3 (9.9)	56.5 (9.8)	56.9 (9.7)	58.3 (9.7)	57.9 (8.4)	56.7 (9.7)	1.93	51,392	0.086	1.21	5, 1176	0.303
Schematization	52.6 (10.4)	54.5 (9.1)	54.8 (9.5)	55.1 (8.7)	54.6 (9.1)	53.4 (10.7)	2.22	51,392	0.050	1.42	5, 1176	0.215
Calculation	53.0 (9.8)	53.8 (9.8)	53.3 (9.7)	55.1 (8.9)	54.3 (10.0)	52.2 (10.8)	2.43	51,392	0.033*	2.04	5, 1176	0.071
Spatial perception	56.0 (10.3)	56.8 (10.1)	58.4 (10.2)	57.6 (10.8)	56.8 (10.4)	55.3 (11.7)	1.90	51,392	0.091	1.02	5, 1176	0.404
Reasoning	56.1 (10.3)	55.2 (11.2)	56.4 (12.6)	56.6 (9.9)	56.1 (10.8)	54.1 (12.4)	1.31	51,392	0.257	0.75	5, 1176	0.589

^a^Items that remained significant after multiple comparison adjustments with a false discovery rate threshold of 0.05 among the items with a *p*-value of < 0.05

The adjusted model included children’s sex, age, gestational age, birth weight, parents’ education level, and household income as covariates

* *p* < 0.05; ** *p* < 0.01

Language development at T4 (38.7 months) assessed with REVT presented significant differences among the six groups on the expressive language subscale (*F* = 3.74; *p* = 0.002), with a higher performance in children breastfed for > 3 months than children breastfed for ≤3 months. However, scores on the receptive language subscale did not differ significantly among the groups.

The performance at T9 assessed by M-FIT showed no significant differences among the six groups. Despite significant differences in the calculation subscale (F = 2.43; *p* = 0.033), the significance was lost after adjusting for covariates and multiple comparisons.

## Discussion

### Prevalence of breastfeeding

The present study investigated the association between breastfeeding and cognitive function in children from 5.5 months to 8 years of age using multiple assessment tools and a prospective design. The prevalence of breastfeeding in our study is comparable to previous studies. Despite evidence of the beneficial effects of breastfeeding on the health of mother and child, the prevalence of breastfeeding was substantially different between countries, with a clear tendency of lower breastfeeding duration and prevalence in wealthier countries [1]. For instance, the proportion of children who were ever-breastfed in our study was 97.4%. The previously reported proportion of ever-breastfed children in most countries was over 90% and was especially high in low-income countries. However, some high-income countries such as France (63%), Spain (77%), Ireland (55%), and the United States (79%) had substantially lower proportions of ever-breastfed children [1]. The proportion of children who continued breastfeeding after 6 months was 61.8% in our study. The average proportion of children who continued breastfeeding after 6 months was lower than 50% in high-income countries, with especially low proportions in Denmark (13%), France (23%), Canada (30%), and the United Kingdom (34%) [1]. The previously reported proportion of breastfeeding at 6 months in Korea was 61%, which is consistent with the present findings [21]. The prevalence of breastfeeding in Korea is reported to have increased remarkably since the lowest prevalence in 2000, which is encouraging news for the health of children [21]. Moreover, the infant mortality rate decreased markedly from 9.9 children per 1000 live births in 1993 to 3.2 in 2009, despite the lack of direct association between increased prevalence of breastfeeding and decreased infant mortality rate [22].

### Early development

Early development of infants at T1 (5.5 months), T2 (14.1 months), and T3 (26.2 months) assessed by Denver II showed no significant differences in odds ratios for developmental delay between the groups of breastfeeding duration. These are inconsistent findings with previous studies. Barros et al. reported significantly higher suspected developmental delay at the age of 1-year assessment in children breastfed for < 1 month (42.4%) compared to those breastfed for ≥9 months (25.5%) [23]. Wang and Wu [24] also reported significantly higher developmental delay in the persona–social domain of Denver II assessed at 1 year of age in non-exclusively breastfed children (36%) compared to exclusively breastfed children (21%).

The results of the early development assessment with K-ASQ presented different aspects than Denver II assessment. The odds ratios to have atypical scores in at least one subdomain of K-ASQ at T2 (14.1 months) was significantly higher by 2.63-fold in children breastfed for 1–3 months than the reference group (children breastfed for 3–6 months). However, there were no significant differences in odds ratios for developmental delay assessed with K-ASQ at T1 (5.5 months) and T3 (26.2 months).

In the comparison of the K-ASQ score as a continuous variable among breastfeeding groups, scores on communication and problem-solving subdomains at T2 (14.1 months) and T3 (26.2 months) in children breastfed for > 3 months were significantly higher than the children breastfed for ≤3 months. These are consistent with the findings of previous studies on early development using the ASQ, which have reported the benefits of breastfeeding on early development. An Irish study of 11,134 children that assessed early development with the ASQ at 9 months old reported the positive effect of breastfeeding on gross motor, fine motor, problem-solving and personal–social skills [25]. A French study with 1999 3-year-old children also reported that ever-breastfed children scored 6.2 points higher on the ASQ than never-breastfed children [26]. The study also reported a significant positive association between exclusively breastfed infants and higher scores on the problem-solving domain of the ASQ. An Australian cohort study with 2868 children reported that infants breastfed for ≥4 months had higher scores in fine motor skills and communication assessed at ages of 1 and 3 years. Infants who were breastfed for < 4 months were also more likely to have at least one atypical score across the subdomains compared to children breastfed for ≥4 months [27].

### Cognitive function in middle childhood and at school age

There were significant differences in cognitive function assessed using the vocabulary test (REVT) among the groups of breastfeeding duration. There was no difference in receptive language score among the six groups of breastfeeding duration. However, when grouped by children who were breastfed for > 3 months or ≤ 3 months, those breastfed for > 3 months scored significantly higher on the vocabulary test. This is consistent with previous findings for language development in middle childhood based on breastfeeding duration. An Australian cohort study with 1195 children assessed language ability with the Peabody Picture Vocabulary Test (mean = 100; SD = 15) and reported that children who were breastfed for > 6 months presented higher mean scores (3.56 points at 5 years and 4.04 points at 10 years, respectively) than children who were never-breastfed [28].

Results for the association between breastfeeding and cognitive function during school days were not significant. There was a significant difference in the scores on the M-FIT subscale of calculation in the comparison of six groups and scores on the vocabulary and language inference in the comparison of two groups (children breastfed for > 3 months versus ≤3 months), with a favorable outcome due to longer breastfeeding duration on the cognitive development. However, the significance was lost after adjusting for confounding variables. These findings are comparable with previous studies which reported the positive associations between the cognitive function of school-age children and breastfeeding duration [29–31]. For instance, children born preterm who were breastfed had higher IQ scores by 7.6 points (about half a standard deviation) at 8 years than never-breastfed children [24]. An Irish study with 8226 9-year-old school children also reported that ever-breastfed children scored significantly higher percentage points on reading and mathematics than never-breastfed children [31]. Huang et al. also reported that breastfeeding had a significant association with higher intelligence and that the association remained significant during the schooling and adolescent period [32].

### Limitations

The present study has some limitations to note. First, due to the characteristics of longitudinal cohort studies, a substantial number of subjects did not participate in the follow-up assessments. Notably, some participants were excluded from the adjusted model analysis due to missing covariate data. The missing data may bias the relationship between breastfeeding and children’s cognitive function. Thus, future study of a more complete dataset with covariate analysis is warranted. Second, although we tried to include important sociodemographic covariates, all covariates could not be included. For instance, previous studies indicated that maternal IQ is a major moderating factor for the association between breastfeeding and children’s intelligence, which was not included in our study [6, 33]. In addition, previous studies included two extents of breastfeeding in their analysis: “any” or “exclusive.” However, we did not collect such information on the extent of breastfeeding. Thus, future studies are warranted to include more detailed information on the related demographic variables and extent of breastfeeding to confirm our findings. Third, our study included many multiple comparisons due to various outcome assessments. Moreover, some significant findings were insignificant after multiple comparison adjustments. Despite these insignificant levels, associations contradictory to our main findings between the prevalence of breastfeeding and delayed development were observed in some development assessments (e.g., lower odds ratio in children breastfed for a lesser period with development delay in Denver II assessment at T1 and T3). The significance level may be influenced by sample number or size of the differences in outcome variables. Thus, although the sample size of our study is not small, a future study with a larger sample size would provide further information to confirm our findings. Despite these limitations, the present study has the strength of using multiple tools at multiple time points to assess children’s cognitive development using a prospective design.

## Conclusion

The findings of our study present a generally positive association between breastfeeding and cognitive function from early childhood through to school age. In contrast, development assessed with some tools (i.e., Denver II) and at some points (T1–5.5 months) revealed null findings for the association. Many previous studies support the finding that there are positive associations between breastfeeding and cognitive development. However, the mean difference (effect size) in cognitive development due to breastfeeding was only 3.44 points (about one-third of a standard deviation), which is reduced again by the adjustment for maternal IQ [6]. Considering these findings comprehensively, breastfeeding is not considered a critical factor in the cognitive development of children. Other studies have also reported that the observed advantage of breastfeeding on IQ score is actually due to genetic and socioenvironmental factors. When the results are adjusted for covariates such as maternal IQ, the effect of breastfeeding on cognitive function was insignificant [34, 35]. Thus, breastfeeding should not be interpreted to have medical benefits for cognitive development. Another study on 12-year-old twins stratified by maternal education level reported a significant effect of breastfeeding on cognition in all strata of maternal education level, although much of the individual difference in cognition scores was accounted for by genetic factors (80%) [33]. Although the reported effects are not significant, it is worthwhile to continue breastfeeding for the possible beneficial effect on children’s cognitive development. In addition, more research to investigate the underlying mechanism for the association between breastfeeding and cognitive development is warranted.

## Supplementary information

**Additional file 1: Supplement Table S1**. Regional distribution of the participants. **Supplement Table S2**. Comparison of scores on intellectual function tests between children grouped based on breastfeeding duration.

## Data Availability

The datasets used and/or analyzed during the current study are available from the corresponding author on reasonable request.

## Abbreviations

ANCOVA: Analysis of variance and analysis of covariance
ASQ: Ages and Stages Questionnaire
K-ASQ: Korean Ages and Stages Questionnaire
M-FIT: Multifactorial intelligence test
PSKC: Panel Study on Korean Children
REVT: Receptive and Expressive Vocabulary Test
