# Supplementary material for: Associations between breastfeeding and cognitive function in children from early childhood to school age: a prospective birth cohort study
Source: Int Breastfeed J. 2020 Sep 29;15:83. doi: 10.1186/s13006-020-00326-4 (PMC7526146; doi:10.1186/s13006-020-00326-4)
Supplement: Supplementary file 1 — Additional file 1: Supplement Table S1. Regional distribution of the participants. Supplement Table S2. Comparison of scores on intellectual function tests between children grouped based on breastfeeding duration. [file 13006_2020_326_MOESM1_ESM.docx]

| Supplement Table S1. Regional distribution of the participants | | | | | | | | |
| --- | --- | --- | --- | --- | --- | --- | --- | --- |
| Region | Excluded participants | | Included participants | | Total | | National population in 2008 | |
|  | n | % | n | % | n | % | n | % |
| Seoul | 66 | 16.6% | 268 | 15.3% | 334 | 15.5% | 10,196,769 | 20.6% |
| Busan | 30 | 7.6% | 107 | 6.1% | 137 | 6.4% | 3,576,008 | 7.2% |
| Daegu | 27 | 6.8% | 124 | 7.1% | 151 | 7.0% | 2,492,993 | 5.0% |
| Incheon | 13 | 3.3% | 48 | 2.7% | 61 | 2.8% | 2,678,636 | 5.4% |
| Gwangju | 14 | 3.5% | 56 | 3.2% | 70 | 3.3% | 1,418,073 | 2.9% |
| Daejeon | 8 | 2.0% | 70 | 4.0% | 78 | 3.6% | 1,478,277 | 3.0% |
| Ulsan | 21 | 5.3% | 84 | 4.8% | 105 | 4.9% | 1,106,201 | 2.2% |
| Gyeonggi-do | 124 | 31.2% | 498 | 28.4% | 622 | 28.9% | 11,199,238 | 22.7% |
| Gangwon-do | 6 | 1.5% | 24 | 1.4% | 30 | 1.4% | 1,506,191 | 3.0% |
| Chungcheongbuk-do | 10 | 2.5% | 57 | 3.3% | 67 | 3.1% | 1,513,098 | 3.1% |
| Chungcheongnam-do | 9 | 2.3% | 98 | 5.6% | 107 | 5.0% | 2,007,034 | 4.1% |
| Gyeongsangbuk-do | 12 | 3.0% | 84 | 4.8% | 96 | 4.5% | 1,859,025 | 3.8% |
| Gyeongsangnam-do | 28 | 7.1% | 101 | 5.8% | 129 | 6.0% | 1,924,418 | 3.9% |
| Jeollabuk-do | 14 | 3.5% | 38 | 2.2% | 52 | 2.4% | 2,677,648 | 5.4% |
| Jeollanam-do | 15 | 3.8% | 95 | 5.4% | 110 | 5.1% | 3,211,104 | 6.5% |
| Jeju-do | - | - | - | - | - | - | 559,938 | 1.1% |
| Total | 397 | 100.0% | 1752 | 100.0% | 2149 | 100.0% | 49,404,648 | 100.0% |
| Statistics for the composition of participants (χ2 = 21.6; p = 0.087) | | | | |  |  |  |  |

| Supplement Table S2. Comparison of scores on intellectual function tests between children grouped based on breastfeeding duration. | | | | | | | | |  | |
| --- | --- | --- | --- | --- | --- | --- | --- | --- | --- | --- |
| Breastfeeding duration | 3 months  or less | more than  3 months | Crude | | | Adjusted | | | | Cohen's d^a^ |
|  |  |  | F | *df* | p | F | *df* | p | |  |
| K-ASQ at T1 (n = 1632) |  |  |  |  |  |  |  |  | |  |
| Communication | 53.7 (8.9) | 53.8 (8.8) | 0.03 | 1, 1630 | 0.855 | 0.01 | 1, 1418 | 0.922 | |  |
| Fine motor | 54.3 (9.0) | 54.9 (8.6) | 2.15 | 1, 1630 | 0.143 | 1.32 | 1, 1418 | 0.252 | |  |
| Gross motor | 57.3 (6.6) | 57.5 (6.2) | 0.23 | 1, 1630 | 0.632 | 0.14 | 1, 1418 | 0.707 | |  |
| Personal-social | 54.2 (8.2) | 54.9 (8.1) | 2.11 | 1, 1630 | 0.147 | 0.44 | 1, 1418 | 0.506 | |  |
| Problem solving | 55.9 (7.3) | 56.1 (7.4) | 0.29 | 1, 1630 | 0.591 | 0.00 | 1, 1418 | 0.978 | |  |
| K-ASQ at T2 (n = 1704) |  |  |  |  |  |  |  |  | |  |
| Communication | 47.7 (11.9) | 50.2 (10.7) | 17.71 | 1, 1702 | **<0.001****^,b^ | 8.75 | 1, 1443 | **0.003****^,b^ | | 0.219 |
| Fine motor | 47.0 (12.1) | 48.1 (11.7) | 3.17 | 1, 1702 | 0.075 | 2.13 | 1, 1443 | 0.145 | |  |
| Gross motor | 56.4 (9.2) | 56.5 (8.7) | 0.12 | 1, 1702 | 0.726 | 0.06 | 1, 1443 | 0.811 | |  |
| Personal-social | 52.1 (11.1) | 52.6 (10.9) | 1.01 | 1, 1702 | 0.315 | 1.73 | 1, 1443 | 0.189 | |  |
| Problem solving | 48.1 (13.3) | 50.2 (11.3) | 11.26 | 1, 1702 | **<0.001****^,b^ | 13.18 | 1, 1443 | **<0.001****^,b^ | | 0.175 |
| K-ASQ at T3 (n = 1752) |  |  |  |  |  |  |  |  | |  |
| Communication | 51.9 (11.3) | 53.3 (10.8) | 6.13 | 1, 1750 | **0.013*** | 4.75 | 1, 1470 | **0.029*** | | 0.127 |
| Fine motor | 52.6 (9.8) | 53.6 (8.4) | 4.17 | 1, 1750 | 0.041 | 2.54 | 1, 1470 | 0.111 | |  |
| Gross motor | 57.2 (6.7) | 57.3 (5.6) | 0.12 | 1, 1750 | 0.727 | 0.12 | 1, 1470 | 0.731 | |  |
| Personal-social | 54.3 (9.5) | 55.1 (8.5) | 2.52 | 1, 1750 | 0.113 | 0.28 | 1, 1470 | 0.597 | |  |
| Problem solving | 53.4 (8.2) | 54.4 (7.6) | 6.79 | 1, 1750 | **0.009****^,b^ | 4.18 | 1, 1470 | **0.041*** | | 0.134 |
| REVT at T4 (n = 1630) |  |  |  |  |  |  |  |  | |  |
| Expressive | 4.4 (3.1) | 5.0 (3.2) | 12.85 | 1, 1628 | **<0.001****^,b^ | 5.55 | 1, 1377 | **0.019*** | | 0.191 |
| Receptive | 6.5 (3.6) | 6.4 (3.6) | 0.00 | 1, 1623 | 0.983 | 0.11 | 1, 1374 | 0.737 | |  |
| M-FIT at T9 (n = 1398) |  |  |  |  |  |  |  |  | |  |
| Vocabulary | 55.4 (10.5) | 57.0 (10.6) | 6.78 | 1, 1396 | **0.009****^,b^ | 1.91 | 1, 1180 | 0.167 | | 0.151 |
| Language inference | 56.4 (9.8) | 57.7 (9.3) | 5.62 | 1, 1396 | **0.018*** | 1.24 | 1, 1180 | 0.266 | | 0.137 |
| Schematization | 53.6 (9.8) | 54.7 (9.3) | 3.73 | 1, 1396 | 0.054 | 0.56 | 1, 1180 | 0.453 | |  |
| Calculation | 53.4 (9.8) | 54.1 (9.7) | 1.43 | 1, 1396 | 0.232 | 0.28 | 1, 1180 | 0.600 | |  |
| Spatial perception | 56.4 (10.2) | 57.1 (10.7) | 1.48 | 1, 1396 | 0.223 | 0.32 | 1, 1180 | 0.572 | |  |
| Reasoning | 55.6 (10.8) | 56.1 (11.1) | 0.58 | 1, 1396 | 0.447 | 0.41 | 1, 1180 | 0.523 | |  |
| a: Cohen's *d* was calculated by the crude model without consideration of covariates.  The number of samples corresponds to the crude model.  The adjusted model included children’s sex, gestational age, birth weight, parents’ education level, and household income as covariates.  REVT-E: Receptive and Expressive Vocabulary Test; M-FIT: Multifactorial Intelligence Test; * *p* < 0.05; ** *p* < 0.01 | | | | | | | | | | |
